# Fatal respiratory disease due to a homozygous intronic *ABCA3* mutation: a case report

**DOI:** 10.1186/s13256-016-1027-z

**Published:** 2016-09-26

**Authors:** Harry Pachajoa, Felipe Ruiz-Botero, Luis Enrique Meza-Escobar, Vania Alexandra Villota-Delgado, Adriana Ballesteros, Ivan Padilla, Diana Duarte

**Affiliations:** 1 Universidad Icesi, Centro de investigacion en anomalias congenitas y enfermedades raras (CIACER), Calle 18 No. 122-135 Edificio Valle de Lili, 5to piso, Pance, Cali, Colombia; 2 Universidad del Valle, Departamento Cirugía Plastica, Calle 4b No. 36-00, Cali, Colombia; 3 Fundación Valle del Lili, Av. Simón Bolívar. Cra 98 # 18-49, Cali, Colombia

**Keywords:** ABCA3, Pulmonary surfactants, Birth defect

## Abstract

**Background:**

Pulmonary surfactant is a complex mixture of lipids and proteins. Mutations in surfactant protein-C, surfactant protein-D, and adenosine triphosphate-binding cassette subfamily A member 3 have been related to surfactant dysfunction and neonatal respiratory failure in full-term babies. Adenosine triphosphate-binding cassette subfamily A member 3 facilitates the transfer of lipids to lamellar bodies. We report the case of patient with a homozygous intronic *ABCA3* mutation.

**Case presentation:**

We describe a newborn full-term Colombian baby boy who was the son of non-consanguineous parents of mixed race ancestry (Mestizo), who was delivered with severe respiratory depression. Invasive treatment was unsuccessful and diagnosis was uncertain. Exons 4 and 5 of the *SP-C* gene showed heterozygous Thr138Asn polymorphism and homozygous Asn186Asn polymorphism respectively. At intron 25 at position –98 from exon 26 a homozygous C>T transition mutation was detected in *ABCA3* gene.

**Conclusions:**

The clinical presentation and the histopathological findings of this case are consistent with a case of neonatal respiratory failure due to surfactant deficiency. Analysis of the five coding *SP-C* exons does not support surfactant deficiency. An analysis of the mutation IVS25-98 T was performed and a homozygous mutation responsible for our case’s neonatal respiratory failure was detected. The findings suggest an autosomic recessive pattern of inheritance. Genetic counseling was provided and the relatives are now informed of the recurrence risks and treatment options.

## Background

Pulmonary surfactant is a complex mixture of lipids, primarily dipalmitoylphosphatidylcholine, surfactant proteins (SP-A, SP-B, SP-C, and SP-D), and the protein adenosine triphosphate-binding cassette subfamily A member 3 (ABCA3), produced by type II pneumocytes. Pulmonary surfactant is essential for lowering surface tension at the air–liquid interface to prevent end-expiratory alveolar collapse. Lamellar bodies are dense multilayer secretory organelles found in pneumocytes II which store the surfactant [1]. Mutations in SP-C, SP-D, and ABCA3 have been related to surfactant dysfunction and neonatal respiratory failure (NRF) in full-term babies and interstitial lung disease (ILD) in older children and adults [2].

ABCA3 is a protein expressed predominantly in the lung, localized to the limiting membrane of lamellar bodies of type II pneumocytes. The ABCA3 protein is codified by a single gene located in chromosome 16 which consists of 33 exons [3]. It has been demonstrated that ABCA3 selectively facilitates the transfer of phosphatidylcholine, sphingomyelin, and cholesterol to lamellar bodies [4].

Autosomic recessive mutations in the *ABCA3* gene have been frequently involved in NRF due to surfactant deficiency and some forms of ILD in older children. The majority of these identified mutations are located in the exons or the immediate intron–exon boundaries. A recent article identified an intronic *ABCA3* mutation in one allele and a known disease causing mutation in the other as responsible for NRF in a full-term newborn [5]. We report the case of a full-term baby boy with a homozygous intronic *ABCA3* mutation as the cause of his fatal respiratory disease.

## Case presentation

**Fig. 1:**
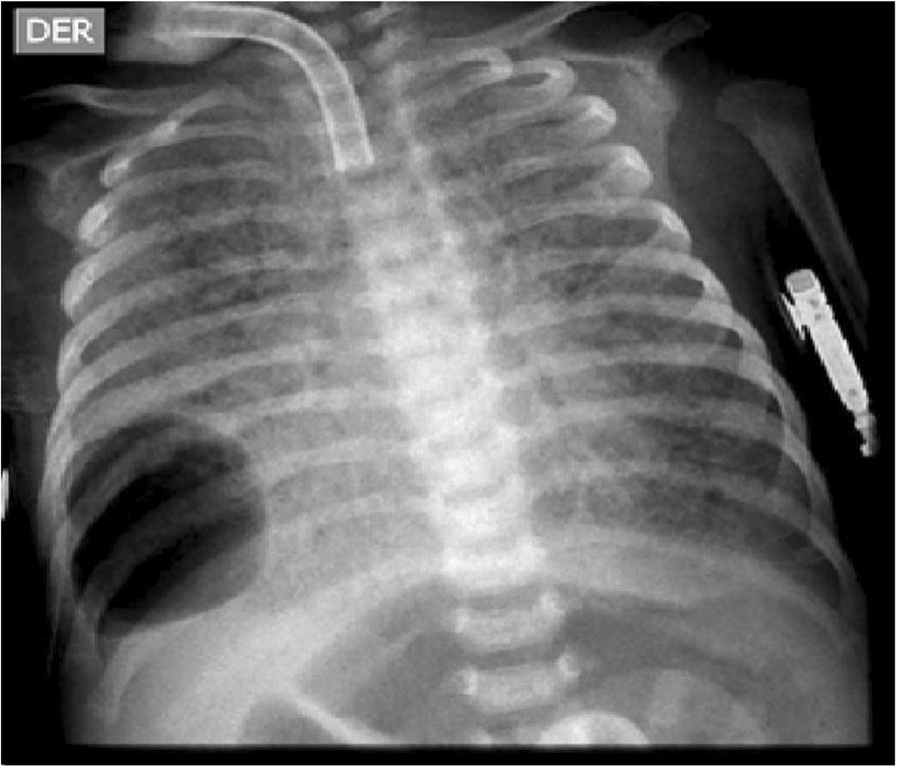
Anteroposterior chest radiograph from a newborn baby boy showing bilateral diffuse hazy granular pulmonary opacification

We describe the case of a full-term Colombian newborn baby boy who was the product of a primigravid mother, and non-consanguineous parents of mixed race ancestry (Mestizo). Fetal monitoring at the 37th week gestational age showed continuous decelerations. A caesarean section was performed and he was delivered with severe respiratory depression. Management with noninvasive positive-pressure ventilation was started without success. He was transferred to our intensive care unit and intubation was performed. An echocardiogram showed moderate pulmonary hypertension. Chest X-rays showed complete bilateral opacity of both lungs. Initial treatment with artificial surfactant was offered without success (Fig. 1).

**Fig. 2:**
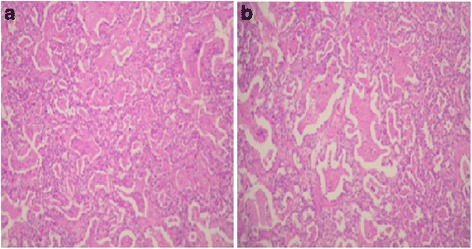
Panels **a** and **b** are different plates from the lung biopsy showing pulmonary alveolar proteinosis pattern characterized by type II pneumocytes hyperplasia, interstitial widening, and fine granular proteinosis material admixed with foamy macrophages

Blood tests showed 38,900 leukocytes with 90 % neutrophils, but the results of all the cultures and the C-reactive protein were negative. A lung biopsy was performed. Right apical pneumothorax appeared as a complication of the procedure and was treated with a chest tube. The lung biopsy showed minimal interstitial changes, preserved alveolar architecture, hyperplasia of the alveolar epithelium (pneumocytes type 2) and a thickened septum full of mesenchymal immature cells and few inflammatory cells (some eosinophils, neutrophils, and leukocytes; Fig. 2).

During his hospitalization, his fever persisted; his leukocytosis was treated with vancomycin and meropenem without any bacteriological finding. He died on day 60 due to respiratory failure and the diagnosis was still uncertain.

Based on the suspicion of a SP deficiency, the genes for SP-B and exon 9 for *ABCA3* gene were analyzed without finding any abnormalities. Exon 4 of the *SP-C* gene showed the polymorphism Thr138Asn in the heterozygous form (ACT/AAT) and exon 5 the Asn186Asn polymorphism in the homozygous form (AAC/AAC).

**Fig. 3:**
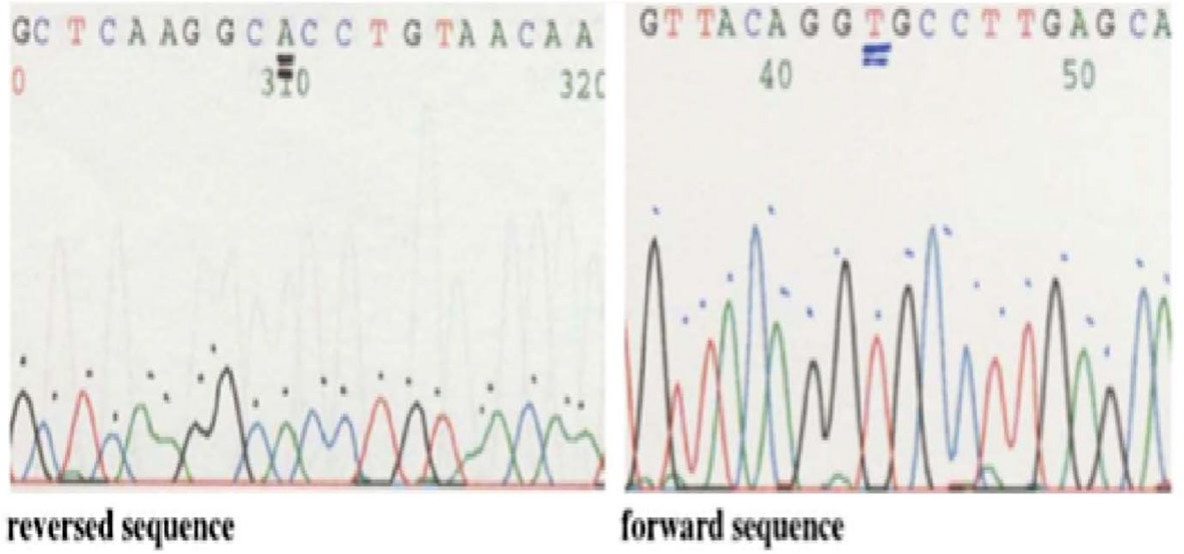
*ABCA3*, exon 26 sequence from 45981 to 46336 showing homozygous mutation IVS25 (gttacagg**T**gccttgag)

Due to the uncertainty of the diagnosis a literature review was performed and experts were asked for advice. A search for a specific mutation in the intronic region of the *ABCA3* gene was performed. In intron 25 at position –98 from exon 26 a homozygous C>T transition mutation was detected. This mutation changes the intronic sequence, creates a new donor splice site and leads to aberrant ABCA3 proteins and is the cause of our patient’s fatal respiratory disease (Fig. 3).

To confirm the mechanism of inheritance of the disease and to be able to perform proper genetic counseling, genetic sequencing for the specific gene was done on both parents and both are carriers of the mutation IVS25-98 T.

## Discussion

The most common presentation of a baby with *ABCA3* mutation that leads to NRF is a full-term baby with moderate to severe respiratory distress and signs of diffuse lung disease without satisfactory history or laboratory findings. The disease is often progressive and fatal within the first 3 months of life even with proper therapy as occurred in this case [6–14]. Some cases of older children with ILD and *ABCA3* mutations have been reported. Out of the four reported cases of patients with a IVS25-98 T mutation in one allele, two died, one of them received a lung transplant and the other one is still alive but has ILD (see Table 1) [5, 7–14].

**Table 1:** Characteristics of patients with *ABCA3* mutation

Author	Patient	Ethnicity	Presentation	Allele 1 mutation	Allele 2 mutation	Findings consistent with ABCA3 deficiency	Outcome	Genotype
Young *et al*. 2008 [7]			15 y/o, indolent exercise intolerance, and chest discomfort	c1–28>G	?		Alive; no clinical, physiologic, or radiographic progression	Heterozygous for *ABCA3*
				IVS9 + 11C>T	?			
				c3765C>G	?			
Hofmeister *et al*. 2008 [8]	Proband	African	Newborn, respiratory distress syndrome	578C>G	578C>G		Died	Homozygous for *ABCA3*
	Proband’s brother	African	Newborn, respiratory distress syndrome	578C>G	578C>G		Died	Homozygous for *ABCA3*
Agrawal *et al*. 2012 [5]	A	White	Newborn, respiratory distress syndrome	p.E690K	IVS25-98T	Lung histopathology and electron microscopy	Died	
	B	White	Respiratory distress syndrome	p.L941P	IVS25-98T	Family history of sibling with fatal respiratory distress syndrome	Died	Heterozygous for *ABCA3*
	C	White	8 y/o, interstitial lung disease	L212M	?	Mutation associated with disease in other patients	Alive with interstitial lung disease	
	D	White	Newborn, respiratory distress syndrome	c.4903ins5	?	Family history of two siblings with fatal respiratory distress syndrome, lung histopathology, and electron microscopy	Died	
	E, F	White	Newborn, respiratory distress syndrome	p.E1325K	?		Died	
	G	Hispanic	2 months, interstitial lung disease	p.R43C	IVS25-98T	Lung histopathology and electron microscopy	Lung transplant	Heterozygous for *ABCA3* IVS25-98C>T
	H	Hispanic	Newborn, respiratory distress syndrome	p.A1070T	?	Mutation associated with disease in other patients, lung histopathology	Alive with interstitial lung disease	
	I	White	Newborn, respiratory distress syndrome	p.R43H	IVS25-98T	Mutation associated with disease in other patients, lung histopathology	Alive with interstitial lung disease	Heterozygous for *ABCA3*
	J	African-American	Interstitial lung disease	p.R280C	?	Mutation associated with disease in other patients, lung histopathology	Alive with interstitial lung disease	
	K	White	interstitial lung disease	p.N1418S	?	Mutation associated with disease in other patients	Alive with interstitial lung disease	
Thavagnanam *et al*. 2013 [9]			Newborn, mild respiratory distress syndrome	c.447 + 11C>T	c.2333 A>G	Family history of sibling with fatal respiratory distress syndrome, lung histopathology, and electron microscopy	Alive	Four variants for *ABCA3*
				c.4583 C>T	c.3755 T>C			
Gonçalves *et al*. 2013 [10]			Newborn, respiratory distress syndrome	L798P	R1612P	Lung histology	Died	Compound heterozygous mutations in *ABCA3*
Panigrahy *et al*. 2014 [11]			Newborn, respiratory distress syndrome	c3703 + 1 G>T	c3703 + 1 G>T	Lung histology	Died	Homozygous for *ABCA3*
Malý *et al*. 2014 [12]			Newborn, respiratory distress syndrome	c.3680 T>G	c4289_4290insA		Died	Two compound heterozygous mutations in *ABCA3*
Rezaei *et al*. 2016 [13]			Newborn, respiratory distress syndrome	p.Gly202Arg/G202R	p.Gly202Arg/G202R		Died	Homozygous for *ABCA3*
Ota *et al*. 2016 [14]		Asian	8 y/o, interstitial lung disease, combined pulmonary fibrosis and emphysema, and pulmonary hypertension	p.L34P	p.1203_1204del	High resolution computed tomography	Alive with interstitial lung disease	Heterozygous for *ABCA3*
Current report		Hispanic	Newborn, respiratory distress syndrome	IVS25-98T	IVS25-98T	Mutation associated with disease in other patients, lung histopathology	Died	Homozygous for *ABCA3*

Original table taken from Agrawal *et al*. 2012 [5], and modified by the authors. *?* Unknown mutation, *y/o* year old

The histopathological findings in patients with NRF due to surfactant deficiency consist of type II pneumocytes hyperplasia, interstitial thickening, and prominent foamy macrophages in the airspaces, often embedded in variable amounts of proteinaceous material as found in the biopsy of our case. These findings are the result of an inborn error disrupting surfactant metabolism and function and are nonspecific for any of the SP-B, SP-C and ABCA3 mutations. A molecular diagnosis is needed to determine the specific mutation affecting each case [15, 16].

In exon 4 our patient shows the polymorphism Thr138Asn in the heterozygous form (ACT/AAT) and in exon 5 the Asn186Asn polymorphism in the homozygous form (AAC/AAC). These findings have been associated with risk of perinatal NRF in preterm male newborns. However, our patient was born at term and these polymorphisms have been frequently found in healthy people. Therefore, one may conclude that the analysis of the five coding *SP-C* exons does not support surfactant deficiency or a fatal malfunction of surfactant transport [17].

Definitive diagnosis required examination of DNA for *ABCA3* intronic mutations. The analysis of the mutation IVS25-98 T was performed and a homozygous mutation was detected. This intronic mutation has been previously reported in heterozygous patients with severe NRF. The genetic analysis of such patients showed one allele with the intronic mutation and an exonic mutation in the other one. It is known that the IVS-98 T is a NRF-causing *ABCA3* mutation since the intronic sequence creates a new donor splice site which leads to aberrantly spliced transcripts. It is suggested that the additional amino acids added to the ABCA3 protein alter its intracellular routing, stability, and function [5]. Recently, intronic mutations have been found in cystic fibrosis as disease-causing mutations in patients without a previously identified exonic mutation [18].

The literature reports four additional cases homozygous for *ABCA3* IVS25-98 T. The four babies were unrelated, no history of consanguinity was identified, but all of them came from South America [5]. Although the mechanism of inheritance is still unclear and isodisomic uniparental disomy has been reported for ABCA3 deficiency, we speculate through our findings that the mechanism of inheritance could be autosomic recessive and the ethnical similarities of the cases could suggest a possible founder effect for this population [6]. Further larger population-based studies are required to determine the real frequency of IVS25-98 T in this population.

In spite of the fact that establishing the diagnosis did not alter the outcome of the patient and usually the diagnosis is established after the decease of the patient, it is essential to adequately counsel the parents and family members of the recurrence risk. Our patient’s parents were encouraged to have their *ABCA3* gene screened for the IVS25-98 T mutation. Both parents of our patient are heterozygous carriers of the IVS25-98 T mutation in the *ABCA3* gene. Therefore, children have a chance of 25 % of being affected by lethal ABCA3 deficiency and 50 % of being carriers. Two sisters of our patient’s father are heterozygous carriers of the IVS25-98 T mutation in the *ABCA3* gene. Their partners do not carry the IVS25-98 T mutation, thus children of both couples have a statistical chance of 25 % of being heterozygous carriers of the mutation and none of them will be affected.

## Conclusions

The clinical presentation of this case is consistent with a case of NRF due to surfactant deficiency. The histopathological findings in patients with NRF due to surfactant deficiency are nonspecific for any of the SP-B, SP-C and ABCA3 mutations and a molecular diagnosis is needed. The analysis of the five coding *SP-C* exons does not support surfactant deficiency or a fatal malfunction of surfactant transport due to *SP-C* mutations as the cause for our patient’s symptoms. Without evidence of a previously identified *ABCA3* mutation in an exon or an immediate intron–exon boundary, we conclude that the homozygous IVS25-98 T mutation is responsible for this case’s NRF. The findings of our patient’s relatives suggest an autosomic recessive pattern of inheritance. Genetic counseling was provided and the relatives are now informed of the recurrence risks and treatment options.

## Abbreviations

ABCA3: Adenosine triphosphate-binding cassette subfamily A member 3
ILD: Interstitial lung disease
NRF: Neonatal respiratory failure
SP: Surfactant protein
